# A Metabolomic Approach to Animal Vitreous Humor Topographical Composition: A Pilot Study

**DOI:** 10.1371/journal.pone.0097773

**Published:** 2014-05-20

**Authors:** Emanuela Locci, Paola Scano, Maria Francesca Rosa, Matteo Nioi, Antonio Noto, Luigi Atzori, Roberto Demontis, Fabio De-Giorgio, Ernesto d'Aloja

**Affiliations:** 1 Department of Biomedical Sciences, University of Cagliari, Cagliari, Italy; 2 Department of Chemical and Geological Sciences, University of Cagliari, Cagliari, Italy; 3 Department of Public Health, Clinical and Molecular Medicine – Forensic Science Unit –University of Cagliari, Cagliari, Italy; 4 Department of Surgery, University of Cagliari, Cagliari, Italy; 5 Institute of Public Health, Catholic University, Rome, Italy; University of Palermo, Italy

## Abstract

The purpose of this study was to evaluate the feasibility of a ^1^H-NMR-based metabolomic approach to explore the metabolomic signature of different topographical areas of vitreous humor (VH) in an animal model. Five ocular globes were enucleated from five goats and immediately frozen at −80°C. Once frozen, three of them were sectioned, and four samples corresponding to four different VH areas were collected: the cortical, core, and basal, which was further divided into a superior and an inferior fraction. An additional two samples were collected that were representative of the whole vitreous body. ^1^H-NMR spectra were acquired for twenty-three goat vitreous samples with the aim of characterizing the metabolomic signature of this biofluid and identifying whether any site-specific patterns were present. Multivariate statistical analysis (MVA) of the spectral data were carried out, including Principal Component Analysis (PCA), Hierarchical Cluster Analysis (HCA), and Partial Least Squares Discriminant Analysis (PLS-DA). A unique metabolomic signature belonging to each area was observed. The cortical area was characterized by lactate, glutamine, choline, and its derivatives, N-acetyl groups, creatine, and glycerol; the core area was characterized by glucose, acetate, and s*cyllo*-inositol; and the basal area was characterized by branched-chain amino acids (BCAA), betaine, alanine, ascorbate, lysine, and *myo*-inositol. We propose a speculative approach on the topographic role of these molecules that are mainly responsible for metabolic differences among the as-identified areas. ^1^H-NMR-based metabolomic analysis has shown to be an important tool for investigating the VH. In particular, this approach was able to assess in the samples here analyzed the presence of different functional areas on the basis of a different metabolite distribution.

## Introduction

Over the last years, the vitreous humor (VH) has gained a pivotal role in comprehending and explaining several ocular diseases, and other medical disciplines are even becoming more interested in analyzing this anatomical structure. Forensic science scholars are well aware of the importance of its investigation to identify several causes of death and, more importantly, to estimate the time since death (Post-Mortem Interval; PMI) [1] and to diagnose acute and chronic drug intoxication [2] and other causes of death (such as heat-related deaths, hyperglycemia, and dehydration) [3], [4]. The attention devoted to the VH by the forensic science community is mainly related to the fact that this biological sample is easy to collect even when an autopsy is not required, it is anatomically isolated and well protected from the external space, and it is less prone to exogenous contamination and degradation. Additionally, its chemical changes appear to occur at a slow rate, expanding its applicability to a wider range of time since death (up to 144 hours) [5], [6]. All the analytes studied in the VH to date exhibit a concentration gradient through the blood-retina barrier (BRB) that is obtained and maintained by several high energy-demanding ATP-dependent Na^+^/K^+^ transporters. Recently, the ophthalmic literature has underscored a more complex function of VH than merely “the clear jelly occupying the inside of the eye”, as it is responsible for oxygen regulation and distribution within the eye [7]–[9]. A proteomic investigation suggested that VH composition may reflect site-specific features [10]; the same may be hypothesized of a metabolomic signature. To investigate this latter hypothesis, we performed a metabolomic analysis of VH in an animal model.

Metabolomics can be defined as the study of the complete set of low-molecular-weight metabolites within a biological fluid, i.e., the “metabolome” by multivariate statistical analysis (MVA) of analytical data obtained from platforms such as Nuclear Magnetic Resonance (NMR) spectroscopy. In particular, ^1^H-NMR is very attractive because it is non-selective, requires minimal sample preparation, and detects all hydrogen-containing mobile molecules [11]–[14]. To our knowledge, few studies are based on a metabolomic approach to investigate retinal diseases [15], and even less used high-resolution ^1^H-NMR spectroscopy to study VH [16], [17]. The aim of the present study was to evaluate the feasibility of a ^1^H-NMR-based metabolomic approach to investigate the metabolomic composition of different VH areas in an animal model. The animal model choice was driven by the need of enucleating ocular globes to be sectioned.

## Materials and Methods

### Sample collection and preparation

**Figure 1:**
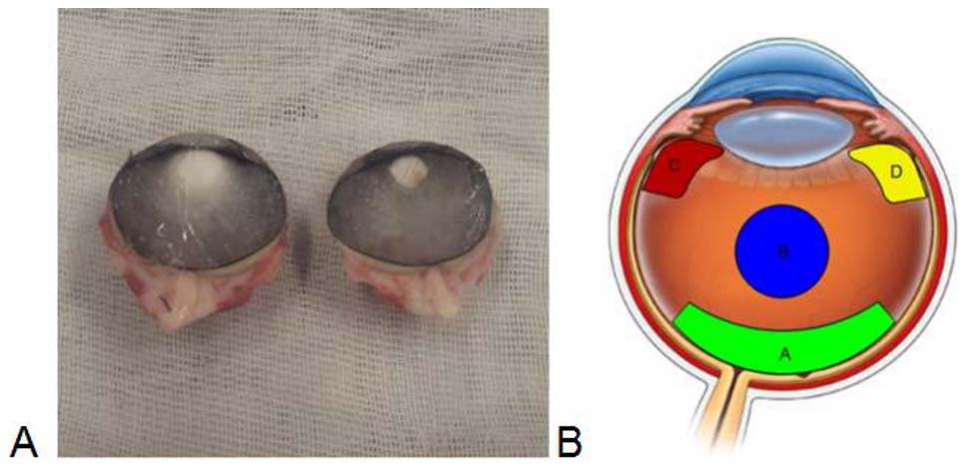
A) An example of a sectioned frozen ocular globe; B) schematic representation of the four VH withdrawal areas: A, B, C, and D.

Goat heads of young adult individuals that passed the standard controls for food consumption were obtained after animal sacrifice from a local slaughterhouse (CO.AL.BE. dei F.lli Contu & C. S.n.c. Selargius, Cagliari, Sardinia, Italy). Generally, goat heads represent waste material, so there were no associated costs. Furthermore, the choice of goat samples was driven by the fact that the ocular globes contain approximately 4 ml VH, which is an adequate amount for ^1^H-NMR analyses of the different topographic areas. In total, five ocular globes were enucleated from five individuals and immediately frozen at −80°C. Subsequently, three ocular globes were sectioned while still frozen using a disposable scalpel through their longitudinal axes (Figure 1A). Four samples of approximately 0.5 ml were obtained using a sterile spatula from each ocular globe (see ref. [10]) corresponding to four different VH areas: the cortical (A), the core (B), and the basal, which was further divided into superior (C) and inferior (D) aliquots (Figure 1B). An additional two whole VH samples (W) were collected. Immediately after collection, each still frozen sample was transferred into an Eppendorf vial and stored at −80°C. The ^1^H-NMR analyses were performed within three months after collection. Before NMR analysis, the samples were thawed and filtered using a 30 kDa filter unit (Microcon-30 kDa; Merck Millipore, Darmstadt, Germany) to remove macromolecules. Glycerol was previously removed from the filters by washing with 500 µl distilled water and centrifuging at room temperature for 10 min at 10000 rpm 15 times. For the NMR analysis, 250 µl filtered vitreous samples were diluted with 350 µl 0.33 M phosphate buffer solution (pH = 7.4) in D_2_O (99.9%, Cambridge Isotope Laboratories Inc., Andover, USA) containing the internal standard sodium 3-trimethylsilyl-propionate-2,2,3,3,-*d_4_* (TSP, 98 atom % D, Sigma-Aldrich, Milan) at a 0.88 mM final concentration and transferred into a 5 mm NMR tube. In total, twenty-three samples were prepared, including nine duplicates.

### ^1^H-NMR experiments

^1^H-NMR experiments were performed on a Varian UNITY INOVA 500 spectrometer (Agilent Technologies, CA, USA) operating at 499.839 MHz. NMR spectra were acquired at 300 K using the standard one-dimensional NOESY pulse sequence for water suppression with a mixing time of 1 ms and a recycle time of 21.5 s. Spectra were recorded with a spectral width of 6000 Hz, a 90° pulse, and 128 scans. Prior to Fourier transformation, the free induction decays (FID) were multiplied by an exponential weighing function that was equivalent to a line broadening of 0.5 Hz and zero-filled to 64 K. All of the spectra were phased, and baselines were corrected using MestReNova software (Version 7.1.2, Mestrelab Research S.L.). 2D NMR ^1^H-^1^H COSY spectra were acquired with a spectral width of 6000 Hz in both dimensions, 4096 data points, and 512 increments with 64 transients per increment.

### Data pre-treatment and multivariate statistical data analysis

Data pre-treatment refers to the methods that are required to make the samples comparable with each other and the overall data suitable for statistical analysis. The ^1^H-NMR spectra were segmented into consecutive integrated spectral regions (bins) of equal width (0.04 ppm) corresponding to the 0.6–8.6 ppm region. The spectral region between 4.20 and 6.08 ppm was excluded from the analysis to remove the effect of variations in the residual water resonance presaturation and adjacent spectral noise. Moreover, because of its disproportionate influence on normalization, the intense doublet that was ascribed to lactate (1.28–1.44 ppm) was also excluded. However, the contribution of lactate to the multivariate statistical models in terms of correlations is retained by the lactate's quadruplet at 4.12 ppm. Binning procedure was performed using MestReNova. The integrated area within each bin was normalized to a constant sum of 100 for each spectrum to minimize the effects of variable concentration among different samples. The final data set consisted of a 23×214 matrix, in which rows represented samples and columns represented the normalized area of each bin (variables). The generated matrix was imported into the SIMCA-P+ program (Version 13.0, Umetrics, Sweden) and Pareto scaled column wise. The multivariate methods employed were (i) unsupervised Principal Components Analysis (PCA) for sample distribution overview; (ii) agglomerative Hierarchical Class Analysis (HCA) to identify sample groups based on the distance among samples; and (iii) the supervised classification technique Partial Least Square Discriminant Analysis (PLS-DA) for the identification of the most discriminant variables that characterize groups. PLS-DA model quality and the optimum number of components were evaluated based on the R^2^ (goodness of fit) and Q^2^ (goodness of prediction) parameters as determined through the default leave-1/7th-out cross validation and tested for overfitting using a y-table permutation test (n = 400). The PCA results were graphically reported in score plots in which samples are projected in the multivariate space. The HCA results, in which the Euclidean distances between samples were measured according to Ward's linkage, were plotted as a tree plot; here, the vertical axis indicates the distance level. Useful parameters obtained from the PLS-DA model were the variable influence on projection (VIP) scores and coefficients that describe the metabolite influence over all of the validated components [18].

## Results

### ^1^H-NMR analysis

**Figure 2:**
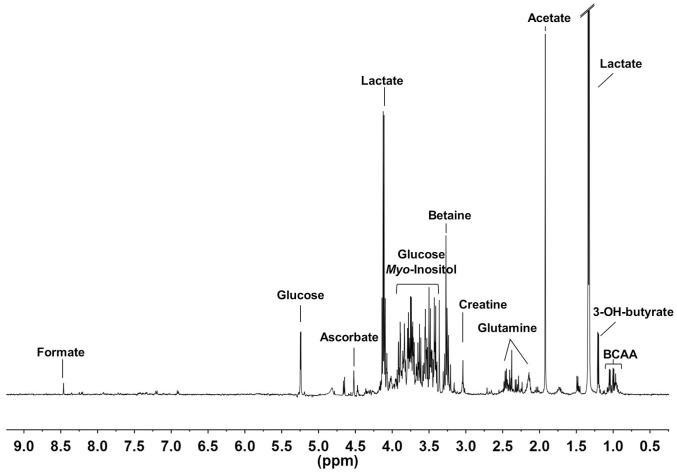
Representative ^1^H-NMR spectrum of a VH sample with main resonance assignments.

The ^1^H-NMR spectra of different VH areas are characterized by sharp peaks assigned to functional groups of low-molecular-weight metabolites found in a free state in the gel-like framework of the VH. Figure 2 shows a representative ^1^H-NMR spectrum of VH samples with major assignments. Assignment of the NMR resonances to metabolites was based mainly on literature data [19], [20], 2D NMR experiments, and the Chenomx NMR suite 7.1 database (Chenomx Inc., Edmonton, Alberta, Canada).

The ^1^H-NMR spectrum can be divided into three main spectral regions. The first (0.5-3.0 ppm) contained signals from the aliphatic groups of free amino acids (FAA), among which are branched chain amino acids (BCAA, i.e., valine, leucine, and isoleucine), glutamine, alanine, and lysine. This region also includes aliphatic signals from organic acids such as lactate, acetate, and 3-OH-butyrate. The second region (3.0–5.5 ppm) exhibits resonances of α-protons of FAA, glucose, glycerol, choline, choline-derivatives (phosphocholine and glycero-phosphocholine), betaine, *myo*-inositol, ascorbate, and creatine. In the spectral region between 6.0 and 9.0 ppm, the less intense signals, attributed to the aromatic protons of tyrosine, phenylalanine, and of nucleosides and nucleobases, such as inosine and hypoxanthine, resonate; the singlet of formate is also visible. Full assignments are reported in Table S1.

A comparative visual analysis of the ^1^H-NMR spectra of the different areas of withdrawal indicates that the samples have common spectral features, except for a higher formate concentration in all of the four topographic regions of Eye 3. To extract the latent information in terms of sample similarities and dissimilarities based on the metabolite characteristics contained in the spectra, we performed MVA.

### MVA

**Figure 3:**
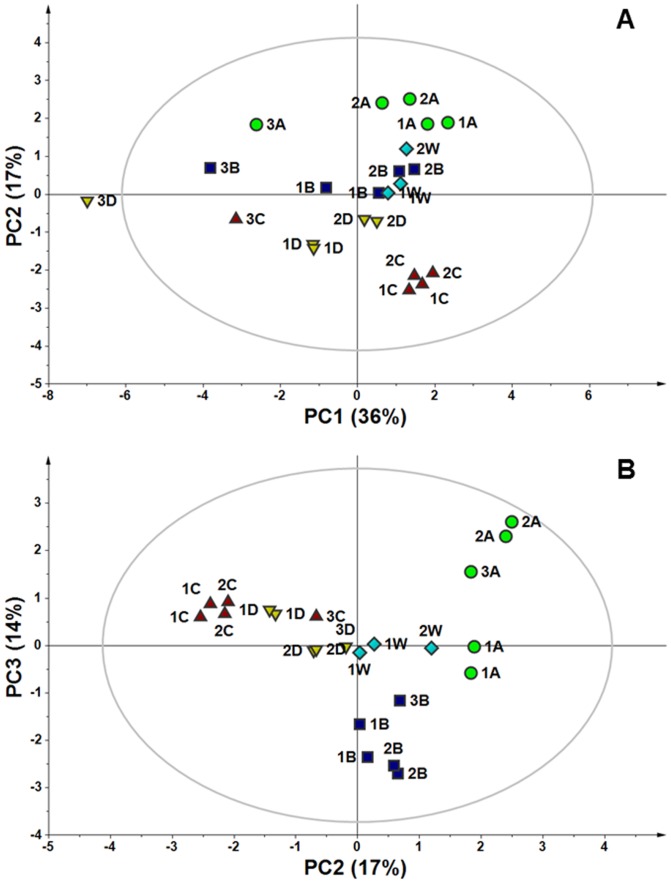
PCA score plots of ^1^H-NMR VH sample spectral data. A) PC1 vs. PC2; B) PC2 vs. PC3. The explained variance is reported in brackets. Numbers represent eye samples, letters represent topographic areas, and W stands for the entire VH (A green circles; B blue squares; C red up-pointing triangles; D yellow down-pointing triangles, W light blue diamonds). Ellipse indicates the 95% Hotelling T^2^ confidence region. Samples with the same number and letter are duplicates.

**Figure 4:**
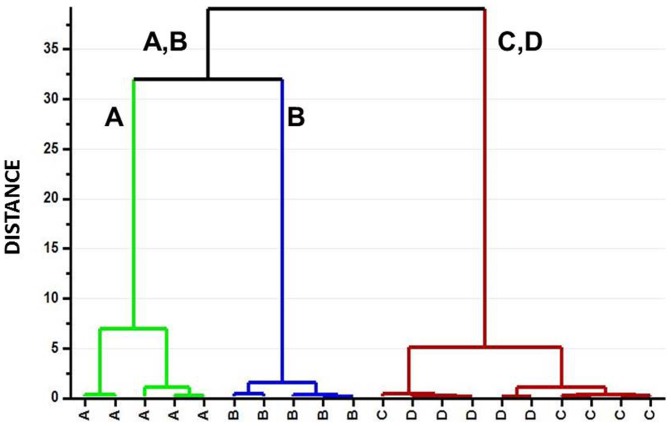
HCA of sample distribution in the PC2 vs. PC3 score space. A tree sorted by Ward clustering. The vertical axis reports sample distances.

**Figure 5:**
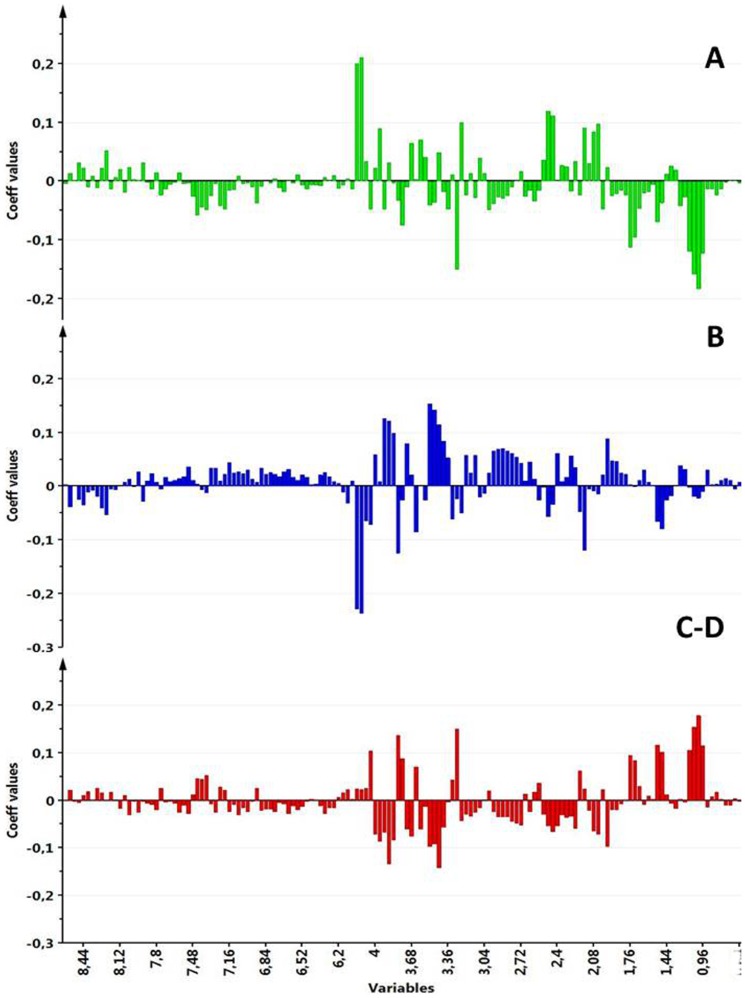
Plot of PLS-DA variable regression coefficients in the three topographical areas: A, B, C–D.

Initially, a PCA was applied to the spectral data. Unsupervised PCA is a tool that provides a multivariate overview of the data based on the underlying variance between the sample metabolite profiles without specifying the different sample types. This type of analysis is useful for screening outliers and for overviewing the tendency of samples to form clusters on the basis of their different spectral features. The first three PCs described 67% of the variance, and all of the samples were within the ellipse of confidence as calculated with the Hotelling T2 test. Analysis of the PC1 vs. PC2 score plot revealed that along PC1 Eye 3 samples were apart from the others, although they maintained the same pattern with regard to the four topographic regions, as shown in Figure 3A. Although NMR visual analysis indicated that formate was the main metabolite responsible for the Eye 3 difference, a careful analysis of the PC1 loadings indicated that other metabolites were involved in the shifting of Eye 3 in the multivariate space. Conversely, the PC2 vs. PC3 score plot (Figure 3B) showed clear tendency of the samples to cluster on the basis of the withdrawal regions. Moreover, the W samples were located almost in the central position of the plot, confirming that they contained average spectral characteristics compared with the other samples. To obtain a measure of the tendency of samples to cluster according to the withdrawal area, HCA was performed on the space spanned by PC2 and PC3 excluding the W samples. Euclidean distances among samples were measured with agglomerative HCA. The results are depicted as a tree plot in Figure 4, where the vertical axis reports the distance between samples. The HCA indicated that the samples clustered into 3 groups: A, B, and C–D. The similarity among samples from the C and D areas is not surprising because both come from the outermost portion of the vitreous, although one was superior and the other was inferior. These overall results demonstrated that the different topographic areas have different metabolite profiles. With the aim of investigating which metabolites characterized the different sample classes, a PLS-DA was performed on the A, B, and C–D classes. PLS-DA is a supervised analysis that maximizes distances between prior defined classes. PLS-DA modeling of the ^1^H-NMR data produced a 3-component model with R^2^(X) = 0.676, R^2^(Y) = 0.883, and Q^2^ = 0.881, indicating good fitting and prediction ability. From the validated model, additional information was obtained from the VIP scores and regression coefficients. Variables with a VIP score larger than 1 were taken into consideration, and the corresponding coefficient values for each topographic area were studied to attribute discriminant metabolites to a specific area (A, B, and C–D). The most significant metabolites characterizing the different areas are reported in Table 1 and are graphically shown in Figure 5. In the latter, the x-axis variables are reported to resemble the ^1^H-NMR spectrum and the y-axis represents the regression coefficient values. It can be clearly observed that the three identified VH areas have different spectral features that correspond to an unequal metabolite distribution. In the cortical vitreous signature (A), the discriminating metabolites are lactate, glutamine, choline and its derivatives, N-acetyl groups, creatine, and glycerol. Glucose, acetate, and s*cyllo*-inositol are characteristic metabolites of the vitreous core (B); in the basal vitreous (C–D), the main distinguished metabolites are BCAA, betaine, alanine, ascorbate, lysine, and *myo*-inositol.

**Table 1:** Variables with VIP >1 and the corresponding regression coefficients for each VH area as calculated by PLS-DA.

Area	Variables	Coefficient values			Metabolites
		A	B	C–D	
**A**	4.12	0.21	−0.24	0.02	Lactate
	4.16	0.20	−0.23	0.02	Lactate
	2.48	0.12	−0.06	−0.05	Glutamine
	2.44	0.11	−0.03	−0.07	Glutamine
	3.24	0.10	−0.05	−0.04	Cho, PCho, GPCho*
	2.04	0.10	−0.01	−0.07	N-Acetyl groups
	2.16	0.09	−0.12	0.02	Glutamine
	3.96	0.09	0.01	−0.09	Creatine
	2.08	0.08	−0.01	−0.06	N-Acetyl groups
	3.6	0.07	0.00	−0.06	Glycerol
**B**	3.52	−0.04	0.15	−0.10	Glucose
	3.48	−0.04	0.14	−0.09	Glucose
	3.92	−0.05	0.13	−0.07	Glucose
	3.88	0.03	0.12	−0.13	Glucose
	3.84	0.00	0.10	−0.08	Glucose
	1.96	0.02	0.09	−0.10	Acetate
	3.4	−0.02	0.08	−0.06	*Scyllo*-inositol
	3.72	−0.01	0.08	−0.06	Glucose
**C–D**	1.00	−0.18	−0.02	0.18	BCAA^†^
	1.04	−0.16	−0.02	0.15	BCAA
	3.28	−0.15	−0.02	0.15	Betaine
	0.96	−0.12	−0.01	0.12	BCAA
	1.52	−0.07	−0.07	0.12	Alanine
	1.08	−0.12	0.00	0.11	BCAA
	4.04	−0.05	−0.07	0.10	Ascorbate
	1.48	−0.04	−0.08	0.10	Alanine
	3.76	−0.08	−0.03	0.09	Ascorbate
	1.76	−0.11	0.00	0.09	Lysine
	1.72	−0.10	0.00	0.08	Lysine
	3.64	0.00	−0.09	0.07	*Myo*-inositol
	3.32	0.01	−0.06	0.04	*Myo*-inositol

Higher regression coefficient values indicate higher comparative levels of the corresponding metabolite in the VH area. Attribution of variables to specific metabolites is also reported.

*Cho (Choline), PCho (Phosphocholine), GPCho (Glycerophosphocholine); ^†^BCAA (Branched Chain Amino Acids).

## Discussion

The approach described herein can identify different metabolomic signatures corresponding to 3 different VH areas (the cortical vitreous, vitreous core, and basal vitreous) [10] because of an uneven metabolite distribution, which is discussed below in terms of their possible functional roles.

### Vitreous cortical area

Lactate is the most represented metabolite of the cortical area. The presence of high lactate levels in human VH has currently been reported using either *in vivo* [21] or *in vitro* [16], [17] NMR spectroscopy. This is because of limited availability of oxygen within the eye, high levels of anaerobic glycolysis, and high oxygen consumption rates in the retina. The relevant insight obtained here is that lactate is topographically more represented in the cortex vitreous area, suggesting that the latter is the most metabolically active. Creatine is a guanidino compound that is synthesized from the amino acids arginine, glycine, and methionine. Its main function is to catalyze the restoration of ATP from ADP, although additional roles such as direct reactive oxygen species (ROS) scavenging are under investigation. Creatine, as well as glycerol, also has an important role in osmo-regulation [22]. Glutamine is the by-product of glutamate conversion by the glutamine synthetase to prevent neurotoxicity of glutamate, which is the main excitatory neurotransmitter in the retina [23], [24]. The characterization of cortical vitreous by means of glutamine may be related to this well-known neuronal survival mechanism. Under normal physiological conditions, astrocytes and Müller glia cells remove excessive glutamate and convert it into glutamine, so the better representation of the latter in the cortical vitreous underlines this metabolic feature. On the basis of these considerations, it can be hypothesized that glutamine plays a protective role in the VH.

### Vitreous core area

Glucose and acetate are among the characterizing metabolites of the core. The former metabolite is the main substrate for ATP production. During life, vitreous glucose levels correspond to the serum concentration, and normal levels in the VH range from 0 to 180 mg/dL. The eye homeostatic capacity maintains a concentration below 100 mg/dL in the VH, even if the glycemia reaches 500 mg/dL [25], [26]. Acetate is an organic acid that is involved in the metabolism of acetylcholine and in the lipid and carbohydrate one. Barba et al. [16] demonstrated that patients with type 1 proliferative diabetic retinopathy presented with acetate accumulation in the VH. A synoptic evaluation of the core vitreous data demonstrated that the molecular picture of this less metabolically involved area is consistent with a passive diffusion of these energetic molecules through the VH area to make them available for the more active areas near the retina.

### Vitreous basal area

In the basal vitreous adjacent to the lens and the trabecular meshwork, the main distinguished metabolites are BCAA, betaine, alanine, ascorbate, lysine, and *myo*-inositol. The crucial role of ascorbate in oxygen regulation and distribution within the eye has been clearly underlined by Holekamp et al. [8]. The vitreous gel, by virtue of its large size and central location within the eye, allows the vascularized retina to be highly oxygenated while protecting tissues that are more sensitive to oxidative stress, such as the lens and the trabecular meshwork. Maintaining a low, even if adequate, oxygen concentration by this ascorbate gradient is important because it preserves lens clarity, which is crystalline in a relatively hypoxic state [9]. As previously described, ascorbate has a differential concentration and is more represented near the cortical vitreous to avoid ROS diffusion from the more active retinal areas towards the anterior segment structures [8]. This result could be explained by uneven distribution of Na^+^-dependent vitamin C transporters (SLC23 family genes SVCT1 and SVCT2, which are more widely represented in the retina). Using ^1^H-NMR analysis, we demonstrated higher ascorbate representation in the basal vitreous. This result may rely on higher SVCT2 density in the distal retina, which would guarantee a higher ascorbate concentration to protect the lens and the trabecular meshwork from oxygen damage.

Osmo-regulator molecules such as betaine and *myo*-inositol also contribute to the basal vitreous metabolic signature. The former is a low-molecular-weight compound that is responsible for cell volume regulation, phosphorylase kinase (PhK) function, and glycogen interaction [27]. The latter is a cyclitol that is naturally present in cells either in its free form or as a bound-component of phospholipids or inositol derivatives. It plays an important role in cellular processes related to survival, development and function of peripheral nerves, osteogenesis, reproduction, and glucose homeostasis [28]. In mammals, *myo*-inositol is present in plasma at concentrations ranging from 25 to 100 µM, whereas intracellular concentrations are several-fold higher, and brain and neuroretinal tissues have the highest concentration [29]. To maintain this gradient, specialized Na^+^-dependent transport systems exist at the BRB to buffer the retina and the VH from plasma oscillations. The main function that can be hypothesized for *myo*-inositol in the VH is related to its role in osmo-regulation, which is a vital task for maintaining retinal structure and function [30]. Hyper- and hypo-osmolar conditions regulate the uptake of several molecules (e.g., taurine, *myo*-inositol, and betaine) together with their osmotically linked water in several cell types including Retinal Pigment Epithelium [30]. The identification of increased *myo*-inositol and betaine levels in the basal vitreous may suggest a peculiar local activity of these molecules focused on preserving the iso-osmotic neuroretina milieu. This function could be regulated by selective transporter (HMIT, H^+^ *myo*-inositol transporter; SMIT1/SMIT2, sodium-dependent *myo*-inositol transporter 1/2) density in the membrane of cells lying in the anterior segment of the eyeball.

Concerning the characterizing presence of BCAA in the basal VH zone, note that BCAA play an important role in the energy and protein metabolism. BCAA in the muscles donate amino groups to furnish glutamic acid; the transamination process of BCAA gives rise to succinate, which enters the citrate cycle and contributes to ATP production by aerobic substrate oxidation [31]. In this view, the contribution of BCAA to the basal VH metabolic signature may be explained as an alternative energetic source to glucose, which is peculiar of the cortical area, as the latter is a more flexible, suitable, and fast response to the functional balance of the high-energy consuming retinal tissue.

In the attempt to provide a general insight into these results, we may postulate that the three identified areas are characterized by a selective distribution of molecules that are mainly involved in energy supply and osmotic regulation. From the first viewpoint, the cortical vitreous relies on a fast glucose-driven metabolic response and its anaerobic pathway, while the basal area is characterized by a relative abundance of BCAA, which may supply energy although in a more time-consuming manner. If observed with an osmo-protector perspective, the basal and the cortical areas are equally involved in tight control of the VH osmotic equilibrium but employ different molecules, namely creatine and betaine/*myo*-inositol.

The finding concerning uneven ascorbate distribution in the VH areas studied here is in agreement with previous reports of differential concentration into the vitreous chamber [8], thus corroborating the hypothesis of a protective role of ascorbate in the lens and trabecular meshwork towards oxygen and ROS.

## Conclusions

The results of this study demonstrate that an uneven topographical metabolite distribution exists in the VH. This finding is consistent with the hypothesis that the site-specific metabolite composition of this biofluid depends on the different locally required functions. This study, although based on a limited animal model sampling, highlights an issue that is well known by ophthalmologists [8]; there is a more complex role of vitreous gel than simply oculogenesis and protection in ocular health and disease. The information gathered in this study may also be useful in forensics, indicating that the metabolomic approach is a powerful tool in this field.

## Supporting Information

Table S1 **^1^H-NMR chemical shifts of the metabolites identified in HV samples.**
(DOCX)
