# Supplementary material for: A Metabolomic Approach to Animal Vitreous Humor Topographical Composition: A Pilot Study
Source: PLoS One. 2014 May 20;9(5):e97773. doi: 10.1371/journal.pone.0097773 (PMC4028277; doi:10.1371/journal.pone.0097773)
Supplement: Table S1 — 1H-NMR chemical shifts of the metabolites identified in HV samples. (DOCX) [file pone.0097773.s001.docx]

Table S1. ^1^H-NMR chemical shifts of the metabolites identified in HV samples.

| **Compound** | **Group** | **^1^H (ppm)^*^** | **^1^H Multiplicity**^†^ |
| --- | --- | --- | --- |
| 3-OH-butyrate | -CH_3_ | 1.20 | d |
|  | -CH_2_ | 2.31, 2.41 | dd, dd |
|  | -CH | 4.15 | m |
| Acetate | -CH_3_ | 1.92 | s |
| Acetoacetate | -CH_3_ | 2.28 | s |
| Alanine | βCH_3_ | 1.48 | d |
|  | αCH | 3.79 | q |
| Ascorbate | -C6H_2_ | 3.75 | m |
|  | -C5H | 4.02 | m |
|  | -C4H | 4.52 | d |
| Betaine | -N-(CH_3_)_3_ | 3.27 | s |
|  | -CH_2_ | 3.90 | s |
| Choline | -N-(CH_3_)_3_ | 3.21 | s |
|  | -O-CH_2_ | 3.51 | m |
|  | -N-CH_2_ | 4.06 | m |
| Creatine | -N-CH_3_ | 3.04 | s |
|  | -N-CH_2_ | 3.93 | s |
| Dimethylamine | -N(CH_3_)_2_ | 2.71 | s |
| Formate | HCOO^-^ | 8.46 | s |
| α-Glucose | -C4H | 3.42 | m |
|  | -C2H | 3.54 | dd |
|  | -C3H | 3.72 | dd |
|  | -C6H2 | 3.76 | m |
|  | -C5H | 3.84 | m |
|  | -C1H | 5.24 | d |
| β-Glucose | -C2H | 3.25 | dd |
|  | -C4H | 3.43 | m |
|  | -C5H | 3.48 | m |
|  | -C3H | 3.50 | t |
|  | -C6H_2_ | 3.90 | dd |
|  | -C1H | 4.66 | d |
| Glutamine | βCH_2_ | 2.15 | m |
|  | γCH_2_ | 2.46 | m |
|  | αCH | 3.78 | t |
| Glycerol | -CH_2_ | 3.57 | dd |
|  | -CH_2_ | 3.66 | dd |
|  | -CH | 3.77 | m |
| Glycerophosphocholine | N-(CH_3_)_3_ | 3.23 | s |
| Glycine | αCH_2_ | 3.57 | s |
| Hypoxanthine (Hyp) | C8H, ring | 8.20 | s |
|  | C2H, ring | 8.22 | s |
| Inosine (Ino) | C1’H, ribose | 6.11 | d |
|  | C2H, ring | 8.35 | s |
|  | C8H, ring | 8.25 | s |
| Isoleucine | αCH | 3.68 | m |
|  | βCH | 1.98 | m |
|  | γCH | 1.45 | m |
|  | γ'CH | 1.27 | m |
|  | γ'CH_3_ | 1.01 | d |
|  | δCH_3_ | 0.94 | t |
| Lactate | βCH_3_ | 1.33 | d |
|  | αCH | 4.12 | q |
| Leucine | αCH | 3.72 | t |
|  | βCH_2_ | 1.70 | m |
|  | γCH | 1.72 | m |
|  | δCH_3_, δ’CH_3_ | 0.96 | d,d |
| Lysine | αCH | 3.75 | t |
|  | βCH_2_ | 1.90 | m |
|  | γCH_2_ | 1.46 | m |
|  | δCH_2_ | 1.72 | m |
|  | δCH_2_ | 3.03 | t |
| *Myo*-Inositol | -C5H | 3.29 | t |
|  | -C1H, -C3H | 3.54 | dd |
|  | -C4H, -C6H | 3.63 | t |
|  | -C2H | 4.07 | t |
| N-acetyl groups | -NHCOCH_3_ | 2.02 – 2.06 | s |
| Phenylalanine | C2,6H, ring | 7.42 | m |
|  | C3,5H, ring | 7.36 | m |
|  | C4H, ring | 7.32 | m |
| Phosphocholine | N-(CH_3_)_3_ | 3.22 | s |
| Pyruvate | -CH_3_ | 2.38 | s |
| Scyllo-Inositol | -CH | 3.36 | s |
| Succinate | -CH_2_ | 2.41 | s |
| Trimethylamine oxide (TMAO) | -O-N-(CH_3_)_3_ | 3.27 | s |
| Tyrosine | C2,6H, ring | 6.91 | d |
|  | C3,5H, ring | 7.20 | d |
| Valine | αCH | 3.61 | d |
|  | βCH | 2.26 | m |
|  | γCH_3_ | 1.00 | d |
|  | γ'CH_3_ | 1.05 | d |

^* 1^H chemical shift are reported with respect to TSP signal (0.00 ppm); ^†^ Multiplicity definitions: s, singlet; d, doublet; t, triplet; q, quartet; dd, doublet of doublets; m, multiplet.
